# Sharp increase in gonorrhoea notifications among young people, EU/EEA, July 2022 to June 2023

**DOI:** 10.2807/1560-7917.ES.2024.29.10.2400113

**Published:** 2024-03-07

**Authors:** Lina Nerlander, Lydia Champezou, Joana Gomes Dias, Gudrun Aspelund, Lina Berlot, Elisavet Constantinou, Asunción Díaz, Jevgenia Epštein, Erika Fogarassy, Victoria Hernando, Patrick Hoffmann, Derval Igoe, Irena Klavs, Pedro Pinto Leite, Kirsi Liitsola, Angeline McIntyre, Zsuzsanna Molnár, Anne Olaug Olsen, Yolanda Pires-Afonso, Renāte Putniņa, Kęstutis Rudaitis, Georgios Siakallis, Sacha de Stoppelaar, Barbara Suligoi, Tuula Hannila-Handelberg, Inga Velicko, Vítor Cabral Veríssimo, Maartje Visser, Maria Wessman, Otilia Mårdh

**Affiliations:** 1 European Centre for Disease Prevention and Control, Stockholm, Sweden; 2 Centre for Health Security and Communicable Disease Control, Directorate of Health, Reykjavik, Iceland; 3 Communicable Diseases Centre, National Institute of Public Health, Ljubljana, Slovenia; 4 Ministry of Health, Nicosia, Cyprus; 5 National Centre of Epidemiology, CIBER in Infectious Diseases (CIBERINFEC), Carlos III Health Institute, Madrid, Spain; 6 Department of Communicable Diseases Epidemiology Health Board, Tallinn, Estonia; 7 National Center for Public Health and Pharmacy, Budapest, Hungary; 8 Health Directorate Luxembourg, Division de l’inspection sanitaire, Luxembourg, Luxembourg; 9 HSE Public Health: National Health Protection Office, Dublin, Ireland; 10 Directorate of Information and Analysis, Directorate-General of Health, Lisbon, Portugal; 11 Finnish Institute for Health and Welfare, Helsinki, Finland; 12 Department of Infection Control and Vaccine, Norwegian Institute of Public Health, Oslo, Norway; 13 The Centre for Disease Prevention and Control, Riga, Latvia; 14 National Public Health Centre, Vilnius, Lithuania; 15 Grigorios HIV Clinic, Larnaca General Hospital, Larnaca, Cyprus; 16 Rijksinstituut voor Volksgezondheid en Milieu, Bilthoven, the Netherlands; 17 National AIDS Unit, Department of Infectious Diseases, Istituto Superiore di Sanità, Rome, Italy; 18 Public Health Agency of Sweden, Stockholm, Sweden; 19 Public Health Unit Cascais, Western Lisbon Local Health Unit, Lisbon, Portugal; 20 Department of Infectious Disease Epidemiology and Prevention Statens Serum Institut, Copenhagen, Denmark

**Keywords:** Gonorrhoea, STI, sexual behaviour, condom use, testing, COVID-19

## Abstract

Gonorrhoea cases increased steeply in women aged 20 to 24 years across 15 EU/EEA countries in July to December 2022 and January to June 2023 with, respectively, 73% and 89% more cases reported than expected, based on historical data from 2015 to 2019. Smaller increases among men due to heterosexual transmission were observed in nine EU/EEA countries. Interventions to raise awareness among young people about sexually transmitted infection risks are needed, emphasising the benefit of safe sexual practices and testing.

In early 2023, four countries in the European Union (EU) and European Economic Area (EEA) (Denmark, Ireland, the Netherlands and Norway) reported increases in gonorrhoea notifications among young people through the EpiPulse and Early Warning and Response platforms coordinated by the European Centre for Disease Prevention and Control (ECDC), The increase started during the second half of 2022 and surpassed the number of cases reported before the COVID 19 pandemic.

Countries in the EU/EEA upload surveillance data for sexually transmitted infections (STI) to the European Surveillance System (TESSy; hosted by the ECDC) by September covering the previous year, including data on gender, age and mode of transmission. In response to the increased rates reported, the ECDC invited countries to submit preliminary data on gonorrhoea diagnoses for the first 6 months of 2023. The main objective was to assess the magnitude of the increase in gonorrhoea cases reported in 2022, including whether this increase continued in early 2023, and to determine in which countries this increase was significant.

## Data sources and analysis

Seventeen countries submitted data from January to June 2023 in addition to the regular upload of 2022 data (Cyprus, Denmark, Estonia, Finland, Hungary, Iceland, Ireland, Italy, Latvia, Lithuania, Luxembourg, the Netherlands, Norway, Portugal, Slovenia, Spain and Sweden).

For the pooled analysis among women, we included data from the 15 countries that had reported consistently for the 5 years before the start of the COVID-19 pandemic in 2020 and in 2022–2023; Spain was excluded because data for 2015 were missing and Luxembourg because of substantial changes in reporting practices. Data from women were included regardless of transmission category (of 35,022 women, 31 399 were reported as heterosexual transmission, 3,190 were unknown or missing and 433 were designated as ‘other'). For the pooled analysis among men, we distinguished between men infected through heterosexual transmission and sex between men, as we expected the trends to be different. We used data from nine countries that reported information on mode of transmission for more than 45% of male gonorrhoea cases for every year in the periods 2015 to 2019 and 2022 to 2023 (Denmark, Finland, Hungary, Ireland, the Netherlands, Norway, Portugal, Slovenia and Sweden).

We described trends in notifications since 2015 for women and trends for men due to either heterosexual transmission or sex between men. We then computed the exceedance above the threshold of cases in 2022 and 2023. Exceedance occurs when the observed number of cases exceeds the expected upper threshold, which we defined as the upper bound of the 95% prediction interval of the expected number of cases from January 2022 to the first half of 2023. It was calculated by applying the Farrington Flexible algorithm [1] to the number of cases among women and among men infected through heterosexual transmission observed during the period January 2015 to December 2019 and forecasting the expected number of cases in 2022 and 2023. The magnitude of the exceedance was determined by calculating the positive differences between the observed value and the threshold. We used the ‘surveillance’ R package [2] to implement the algorithm. We did not include data from 2020 and 2021 for the estimates of the expected number of cases because of the likely impact of reduced transmission, under-ascertainment and under-reporting due to the COVID-19 pandemic (internal ECDC survey 2022, data not shown) [3]. Analyses were done by age group. All cases 15 years and older were included in the first part of the analyses. The exceedance analysis focussed on ages 15–29 years. Country-level analyses were done for women aged 20–24 years for all countries except Luxembourg. 

## Trends observed

**Figure 1:**
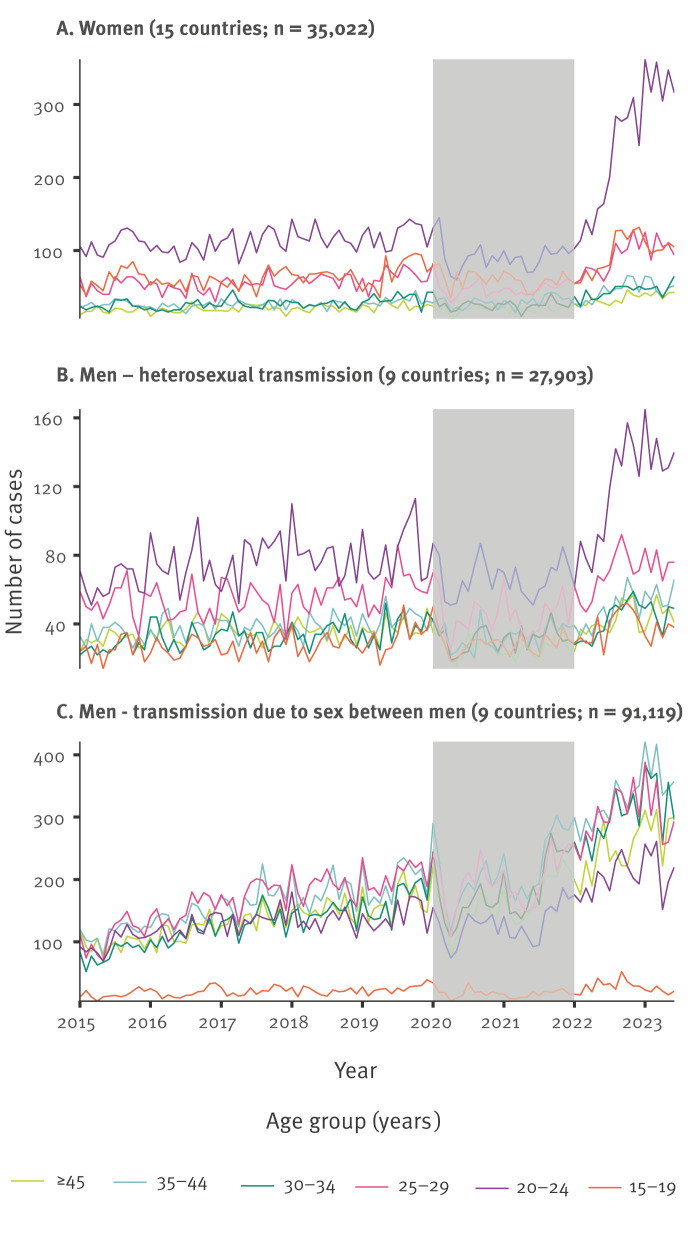
Trends in the number of gonorrhoea cases by month and by age group, EU/EEA countries, January 2015–June 2023 (n = 154,044)

EEA: European Economic Area; EU: European Union.

Among women, the number of reported cases started to rise sharply in mid-2022 among 20–24-year-olds, and to a lesser extent among 15–19-year-olds and 25–29-year-olds (Figure 1). These increases were paralleled by increases due to heterosexual transmission among men in mid-2022 among 20–24-year-olds and 25–29-year-olds (Figure 1). A different pattern was observed among men who report sex between men, where the number of reported cases has been increasing gradually since 2015 across all age groups, except those aged 15–19 years (Figure 1).

The grey area highlights 2020 and 2021 when surveillance data for sexually transmitted infections are likely to have been impacted by reductions in transmission, under-ascertainment and under-reporting due to the COVID-19 pandemic. Countries included for panel A (Women): Cyprus, Denmark, Estonia, Finland, Hungary, Iceland, Ireland, Italy, Latvia, Lithuania, the Netherlands, Norway, Portugal, Slovenia and Sweden. Countries included for panels B and C (Men): Denmark, Finland, Hungary, Ireland, the Netherlands, Norway, Portugal, Slovenia and Sweden.

## Quantifying the exceedance

**Figure 2:**
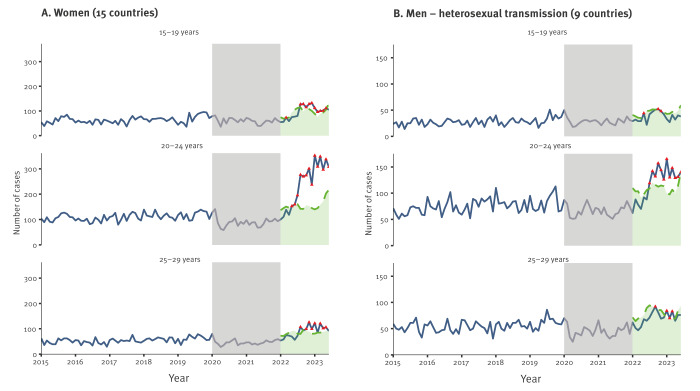
Number of gonorrhoea cases (January 2015–June 2023) compared with the exceedance threshold (January 2022–June 2023), by month, EU/EEA

EEA: European Economic Area; EU: European Union.

The largest exceedance was seen among women aged 20–24 years with 72.6% more reported cases than the expected upper threshold in the second half of 2022 and 88.6% more in the first half of 2023 (Figure 2, Table). In women aged 15–19 years and 25–29 years, the exceedances in reported cases were, respectively, 18.4% and 25.1% in the second half of 2022, and 15.3% and 30.4% in the first half of 2023. The exceedance started in May 2022 among women aged 20–24 years and in August 2022 among 15–19-year-olds and was detected in most months between July 2022 and June 2023. Increases in rates above the expected upper bound were also seen in the older age groups that were not the focus on the exceedance analysis (30 years and older) but the magnitudes were substantially lower compared with the 20–24-year-olds.

Blue line: number of reported (observed) gonorrhoea cases from January 2015 to June 2023. Green dashed line: exceedance threshold as derived from the Farrington Flexible algorithm using historical data from January 2015 to December 2019. The upper threshold limit reflects the 95% percentile of the distribution of expected cases using a negative binomial model. Red triangles: indicate an alarm, whenever the observed cases exceed the upper threshold limit. This means that the observed number of cases differs statistically from the expected number of cases. Grey area: 2020 and 2021 excluded from the historical data due to likely effects of the COVID-19 pandemic on transmission and reporting during this time.

Countries included for panel A (Women): Cyprus, Denmark, Estonia, Finland, Hungary, Iceland, Ireland, Italy, Latvia, Lithuania, the Netherlands, Norway, Portugal, Slovenia and Sweden. Countries included for panel B (Men, due to heterosexual transmission): Denmark, Finland, Hungary, Ireland, the Netherlands, Norway, Portugal, Slovenia and Sweden.

**Table:** Observed cases of gonorrhoea and exceedance for January 2022–June 2023, among women (15 countries) and among men infected through heterosexual transmission (nine countries), EU/EEA (n = 11,872)

	Observed cases Jan 2022–Jun 2023	Number of months above the threshold	Exceedance overall or by 6-month period (n)	Exceedance (%)^a^
**Women^b^** (n = 7,787)				
Age 15–19 years	1,730	11	169	16.0
Jan–Jun 2022	395	1	2	2.8
Jul–Dec 2022	710	5	98	18.4
Jan–Jun 2023	625	5	69	15.3
Age 20–24 years	4,404	14	1,624	70.6
Jan–Jun 2022	802	2	10	3.2
Jul–Dec 2022	1,597	6	672	72.6
Jan–Jun 2023	2,005	6	942	88.6
Age 25–29 years	1,653	8	197	27.8
Jan–Jun 2022	396	0	No exceedance	No exceedance
Jul–Dec 2022	613	4	89	25.1
Jan–Jun 2023	644	4	108	30.4
**Men infected through heterosexual transmission^c^** (n = 4,085)				
Age 15–19 years	678	3	8	5.9
Jan–Jun 2022	185	1	3	7.3
Jul–Dec 2022	282	2	5	5.3
Jan–Jun 2023	211	0	No exceedance	No exceedance
Age 20–24 years	2,141	11	318	26.4
Jan–Jun 2022	478	0	No exceedance	No exceedance
Jul–Dec 2022	820	6	133	19.4
Jan–Jun 2023	843	5	185	35.7
Age 25–29 years	1,266	4	31	10.4
Jan–Jun 2022	346	0	No exceedance	No exceedance
Jul–Dec 2022	466	1	1	1.1
Jan–Jun 2023	454	3	30	14.5

EEA: European Economic Area; EU: European Union.

^a^ The exceedance percentage is computed dividing the sum of exceedance by the sum of the expected upper thresholds for the specific months exceeding this threshold.

^b^ 15 countries: Cyprus, Denmark, Estonia, Finland, Hungary, Iceland, Ireland, Italy, Latvia, Lithuania, the Netherlands, Norway, Portugal, Slovenia and Sweden.

^c^ Nine countries: Denmark, Finland, Hungary, Ireland, the Netherlands, Norway, Portugal, Slovenia and Sweden.

Among young men infected through heterosexual transmission, exceedances were of smaller magnitudes than in young women. Among those aged 20–24 years, exceedances started in July 2022 and were highest in the first half of 2023 (35.7%). Increases in rates above the expected upper bound were also seen in the older age groups (30 years and older) but with lower magnitudes compared with the 20–24-year-olds. The rise in cases among men due to sex between men was not the focus of this part of the analysis as this increase has been gradual since 2015.

## Country-specific results

Country-level analysis focussed on women aged 20–24 years. Exceedances of more than 100% in either the second half of 2022 or the first half of 2023 were seen in Denmark, Iceland, Ireland, the Netherlands, Norway, Portugal and Slovenia. Three countries – Finland, Italy and Sweden – saw exceedances of smaller magnitudes (5–31%). Estonia, Hungary and Latvia did not see any exceedance, and Cyprus and Lithuania did not have enough data for country-level estimations. Based on data from Spain from regions that reported consistently from 2016 onwards, exceedances of 116% and 98% were seen among women aged 20–24 years in the second half of 2022 and the first half of 2023, respectively. Of note, the heterogeneity between the countries’ surveillance systems limits comparisons between countries [4].

## Sensitivity analysis and limitations

In a sensitivity analysis, we examined the exceedances among women, using data from only the nine countries included in the analysis of men, restricted to female cases where the mode of transmission was ‘heterosexual’ (rather than using all female cases in the main analysis where many cases had missing data on mode of transmission but were assumed to be heterosexual). Compared with the main analysis, exceedances were slightly lower for the youngest age group (14% and 9%, respectively, for the second half of 2022 and first half of 2023) and for the second half of 2022 for the 20–24-year-olds (53%).

Several limitations apply to the main analysis and the sensitivity analysis. Data for 2023 are preliminary, and some corrections in the number of cases may occur. Data from 2023 were only available from half of the EU/EEA countries for the analysis among women, and for nine countries for the analysis among men; thus, the geographical extent of these increases is still to be determined.

## Discussion

The sharp increase in gonorrhoea notifications affecting women is unprecedented since 2009 when the ECDC started to coordinate STI surveillance in the EU [5]. The observed increases with a predominance among young women are concerning as untreated *Neisseria gonorrhoea* infection can lead to complications including pelvic inflammatory disease, chronic pain and subsequent infertility [6,7]. The EU/EEA surveillance data for 2022 for chlamydia and syphilis also indicate increases in almost all age groups of women and men. With regards to the development in young people between 2021 and 2022, the number of chlamydia diagnoses per 100,000 women aged 20–24 years increased by 18% and that of syphilis cases by 50%. Among men aged 20–24 years, the chlamydia and syphilis diagnoses increased by 14% and 41%, respectively [8,9]. Future analyses should compare rates of gonorrhoea, syphilis and chlamydia by age and transmission category to understand the extent to which increases are general or specific to gonorrhoea. Recent data from Denmark found a lower relative increase in chlamydia notifications compared with gonorrhoea and genetic lineages of *N. gonorrhoeae* distinct from those seen among men who have sex with men [10].

The ECDC organised a series of meetings where national experts indicated several possible theoretical hypotheses for factors driving these increases. These included changes in sexual behaviours such as number of partners and how sexual partners are connected to each other, sexual practices, condom use [11], travel patterns, and the role of bridging populations such as men who have sex with women and men, given the concurrent increases in infections due to sex between men. Changes in testing policies with expansion of free and easily accessible testing (e.g. online) could have improved case detection in some settings, as could a shift towards more sampling from extra-genital sites. In Ireland, an online service offering free home sampling that had national reach by October 2022 increased access to testing; in 2023, 34% of female cases and 22% of male cases were notified by this system [12] (personal communication: Angeline McIntyre, Health Protection Surveillance Centre, Ireland, February 2024). However, in the Netherlands, the positivity rate among tests has increased, indicating that the increased diagnoses are not only due to increased testing [13]. Another hypothesis raised was that the *N. gonorrhoeae* lineages driving this increase among heterosexual populations may cause no or low-grade symptoms or be more transmissible [10]. It is also possible that the group with the highest increases belongs to a cohort of young people whose social interactions were restricted during the COVID-19 pandemic at an age when their first sexual contacts would normally have taken place. This could have resulted in different sexual behaviours after the pandemic in this group compared with previous cohorts. 

Some countries indicated that they had, or were planning to, initiate investigations into possible drivers for this increase, including behavioural surveys, sexual networks, and molecular typing analyses to define the role of sexual behaviours and bridging populations, identify clusters, and determine geographical spread.

Many countries have already initiated measures in response to the increased gonorrhoea notifications. If not already done, the following actions can be initiated or expanded: raising awareness among young people about the increasing risk of STI, including in educational settings, emphasising condom use as well as testing before or after engaging in unprotected sex, employing innovative approaches (e.g. through social media), ensuring that healthcare providers adhere to evidence-based guidelines, and strengthening partner notification services, along with collecting timely, high-quality surveillance data. Surveillance of antimicrobial resistance and monitoring of treatment failures as done through the Euro-GASP Network are also warranted to detect any emergence of resistant strains and appropriately amend the treatment guidelines. Analysis of gonorrhoea cases in Denmark showed that lineages among heterosexuals were highly susceptible to antibiotics recommended for first line therapy in Denmark [10]. A better understanding is needed of which clades spread within sexual networks, either among heterosexual individuals or men who have sex with men, and of their characteristics.

Research is needed to understand the reasons for the increases. The Irish Health Research Board has put out such a call for evidence [14]. Behavioural surveys would support the understanding of the underlying drivers, and qualitative work can also provide valuable insight into potential changes in sexual behaviour that can allow more targeted interventions. The ECDC will maintain a close dialogue with the EU/EEA countries and will facilitate sharing of results from investigations, the exchange of good practice, and provide scientific and laboratory support.

## Conclusion

The sharp increases in gonorrhoea notifications in young people, particularly women aged 20–24 years are of concern. Immediate actions are needed to raise awareness among young people about the importance of condom use and testing. Further work is needed to understand the factors driving these increases in order to effectively target public health interventions. 
